# Risks Related to Advanced Bridge Monitoring Technologies

**DOI:** 10.3390/s26051603

**Published:** 2026-03-04

**Authors:** Michal Miške, Pasquale Daponte, Luca De Vito, Lucia Figuli

**Affiliations:** 1 Faculty of Security Engineering, University of Žilina, 010 26 Žilina, Slovakia; figuli@uniza.sk; 2 Department of Engineering, University of Sannio, 82100 Benevento, Italy; daponte@unisannio.it (P.D.); devito@unisannio.it (L.D.V.)

**Keywords:** structural health monitoring, risks assessment, infrastructure resilience, cybersecurity, bridge infrastructure, Digital Twin

## Abstract

Bridge monitoring has undergone a significant transformation with the integration of advanced technologies, including structural health monitoring systems, Internet of Things sensors, unmanned aerial vehicles, artificial intelligence, and cloud computing. These technologies enable continuous real-time data acquisition, processing, and early detection of structural degradation. However, their deployment also introduces a range of emerging risks that require careful consideration. This paper presents descriptive risk listings and proposes a comprehensive risk-governance framework for advanced bridge monitoring using the SWOT analysis. The framework integrates a unified risk taxonomy and assessment that links sensor and AI performance with cyber threat modeling and data governance requirements. The application of two real deployments, the Jindo Bridge SHM program and the Stava Bridge digital-twin implementation, shows how the framework converts heterogeneous measurements for improving bridge lifecycle management with the implementation of advanced monitoring technologies. Compared with prior studies that primarily catalog risks, the contribution of the paper is an interdisciplinary, operationalizable method that couples reliability, security, and governance into a single process, thereby ensuring that advanced technologies enhance, rather than erode, the safety and resilience of bridge infrastructure.

## 1. Introduction

**Figure 1:**
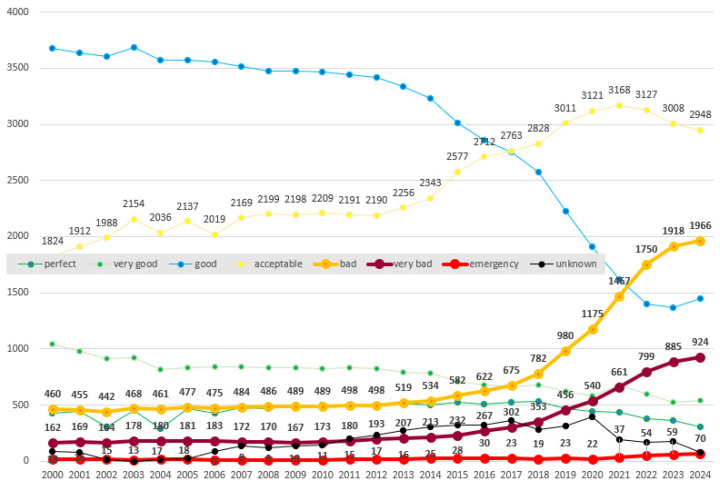
Structural health conditions of Slovak bridges [1].

The aging of bridges, changing climatic conditions, and increasing traffic loads currently necessitate the implementation of effective, reliable, and comprehensive systems for monitoring their structural and technical condition. Many bridges were designed and built in the previous century, often according to now-outdated standards, and their material capacity, structural design, and operational load no longer meet current requirements. Consequently, their condition is gradually deteriorating due to natural aging, climatic influences, and intensive use. Referring to Slovakia as a case study, this degradation is confirmed by statistics from the Slovak Road Administration and the National Road Database, which indicate an increasing number of bridges classified as being in bad, very bad, or emergency condition, as illustrated in Figure 1.

Beyond these structural and environmental drivers, as an example, the Slovak bridge stock is also affected by system weaknesses in the inspection, decision, and intervention chain. Condition assessment has traditionally relied heavily on periodic visual inspections and expert judgment, which is once every 4 years. This can introduce subjectivity, inconsistencies between inspectors, and limited comparability over time. In addition, the absence of continuous monitoring of many assets reduces the ability to detect early-stage degradation or defects (e.g., microcracking, hidden corrosion, fatigue damage) before they become critical. As a result, maintenance decisions are often made reactively, once defects have progressed, rather than proactively based on objective condition indicators. A further aggravating factor is the delayed allocation of financial resources for rehabilitation and renewal, which can lead to postponed interventions, accumulation of maintenance backlogs, and a progressive shift of bridges into worse condition classes. Together, insufficient monitoring coverage, subjective decision-making, and late or inadequate funding contribute to accelerated deterioration and increased safety and operational risks within the Slovak bridge infrastructure network [1].

Traditional diagnostic methods, especially visual inspection carried out by field workers, remain important but are increasingly encountering their limitations. These include time and personnel demands, subjectivity in evaluation, and especially a limited ability to detect hidden defects early, such as microcracks, internal corrosion, or fatigue damage [2,3,4].

**Figure 2:**
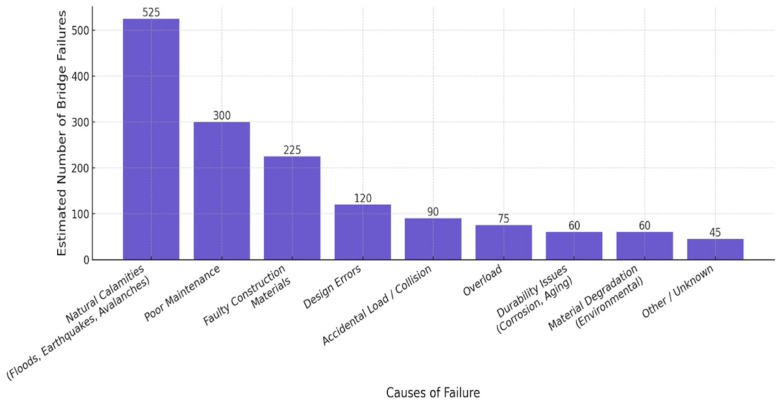
Global classification of bridge failures due to different causes [6].

Inspection of a bridge in challenging terrain, for example, over watercourses or deep gorges, requires specialized equipment, which increases costs, prolongs inspection duration, and increases risks to inspection personnel. Another fundamental problem is the shortage of qualified experts conducting these inspections and assessments. Given the huge number of bridges registered, it is long-term unsustainable for the managing company to ensure their regular and detailed inspection solely by conventional means [1]. In this context, modern technologies are emerging as key, effectively complementing or automating certain phases of bridge monitoring. These include, for example, sensor systems for Structural Health Monitoring (SHM), which provide continuous measurement of key physical parameters of the structure (e.g., stress, vibrations, temperature, humidity); high-resolution drones used for visual inspection of hard-to-reach parts of the structure; or artificial intelligence, which can process large volumes of data and automatically identify defects [5,6]. These technologies provide infrastructure managers with immediate, objective information on the current technical condition of the bridge. As a result, faster, more targeted decision-making is possible regarding necessary interventions, maintenance planning, and preventive safety measures. Early identification of risks also reduces the likelihood of accidents, optimizes operational costs, and extends the service life of the bridge network. On the other hand, infrastructure resilience can be strengthened to mitigate the impact of the most common causes of failure and collapse (illustrated in Figure 2). Figure 2 by Prakash represents global data from the years 1907 to 2024.

**Figure 3:**
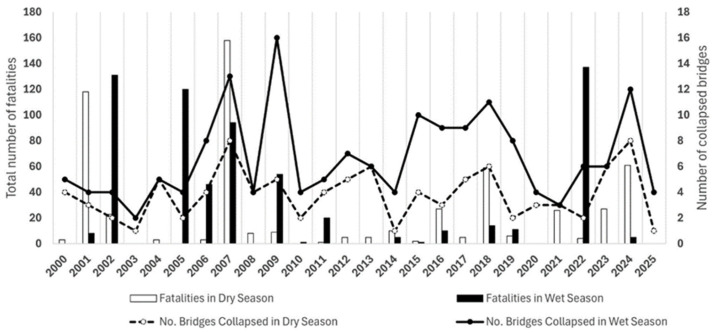
Normalized number of fatalities and number of reported collapsed bridges in each year in the context of seasonal change [10].

Only over the past decade have numerous failures occurred worldwide, followed by an equally large number of fatalities, as shown in Figure 3. These incidents have not only resulted in the loss of lives but also highlighted vulnerabilities in infrastructure. Even in cases where no casualties were reported, the total failure of bridges, often those already closed to traffic, has had a profound impact on local communities and services. Bridges are integral components of transportation infrastructure, serving as crucial links for commuters traveling to work, school, or other essential destinations [7]. When such infrastructure fails, it forces people to take lengthy detours, resulting in delays, increased transportation costs, disruptions to their daily routines, and a higher environmental impact. Furthermore, the extended time required to rebuild or replace these bridges can strain resources and add to the overall economic burden, affecting both individuals and the broader society. The ripple effects of these collapses emphasize the need for a more robust and proactive approach to infrastructure maintenance, assessment, and safety in Europe [8,9].

Advanced bridge monitoring technologies (e.g., SHM systems, IoT-enabled platforms, wireless sensor networks, and geodetic methods) offer substantial benefits, but they also introduce new categories of risk that extend beyond the structure itself to encompass measurement quality, data infrastructure, and result interpretation [11]. In IoT-based environments, critical vulnerabilities include power constraints, communication latency, transmission reliability, and metrological integrity, all of which directly affect data trustworthiness and, consequently, downstream engineering decisions [12]. Lessons learned from long-term SHM deployments further indicate that the core challenge is not merely collecting data but ensuring time synchronization, quality assurance, robust data management, and, most importantly, reliable inference from signals to structural condition, which is also central to the development of cyber-physical systems for infrastructure. Accordingly, building smart SHM platforms for long-span bridges requires a coherent end-to-end data strategy (acquisition–processing–management–analytics–visualization) and an explicit link to decision-making; otherwise, monitoring risks becomes an expensive source of underutilized or poorly comparable datasets [13]. Individual technologies also have domain-specific limitations—for example, high-rate GNSS can provide direct displacement estimates, but its accuracy and stability depend strongly on processing approaches and measurement conditions, which may propagate significant uncertainty into deformation assessments. Finally, large-scale historical field deployments of wireless sensing on real bridges (e.g., the ISHMP/Jindo Bridge testbed) demonstrate the feasibility of such systems while underscoring the need for robust long-term operation, data governance, and interpretation workflows [14,15].

This paper provides a structured state-of-the-art overview of advanced technologies for bridge monitoring with a specific focus on the risks introduced by their deployment. We synthesize recent evidence on UAV/robot-based visual inspection, AI-driven defect detection, SHM/IoT sensing architectures, and Digital Twin concepts [16] and reorganize these technologies along the functional monitoring chain (data acquisition, visual inspection, and intelligent diagnosis) to improve logical continuity from technology description to risk discussion. The main contribution is a monitoring-chain-based risk framing that maps typical failure modes and uncertainties to each stage, offering a clear basis for risk-aware technology selection and for designing more robust monitoring workflows for critical bridge infrastructure [17]. The primary objective of this paper is to highlight alternative perspectives on the implementation of advanced technologies, which are incredibly useful; however, they can also generate new potential risks. Section 2 provides an overview of technologies primarily used for bridge monitoring, including digital twins and the Internet of Things, among others. The third section focuses on SWOT analysis, which clarifies the potential of modern technology in relation to the risks associated with its use. Then, in the following Section 4, a risk matrix is provided where an expert’s estimate quantifies the risks retrieved from using advanced technologies.

## 2. Overview of Technologies

The goal of using advanced technologies is not only to monitor bridges but also to simplify processes, enhance the resilience of the structures, and improve the quality of structural life. Historically, decision-making relied heavily on expert judgment, where the potential for human error or subjective bias could result in undesirable outcomes [3]. This shift from only human experts to experts using advanced technologies reduces blind spots between periodic inspections, accelerates detection of early-stage defects, and supports predictive maintenance that extends service life while minimizing traffic disruptions [10]. The process of monitoring and following assessment of resilience has four main steps: data acquisition, visual inspection, intelligent diagnosis, and preventive measurements. In case of new risks, the process of risk evaluation must be followed (risk identification, risk analysis, and risk evolution). For this paper, the focus will be on the currently most widely used technologies, which are listed in the order in which they enter the process of monitoring the current status and subsequent evaluation, which include:

1. SHM systems for data acquisition;
2. Sensor Networks based on the Internet of Things (IoT) for data acquisition;
3. Unmanned Aerial Vehicles (UAVs—drones) for visual inspection;
4. Artificial Intelligence (AI) for intelligent diagnosis;
5. Digital Twins (DTs) for intelligent diagnosis and preventive measurements.

**Figure 4:**
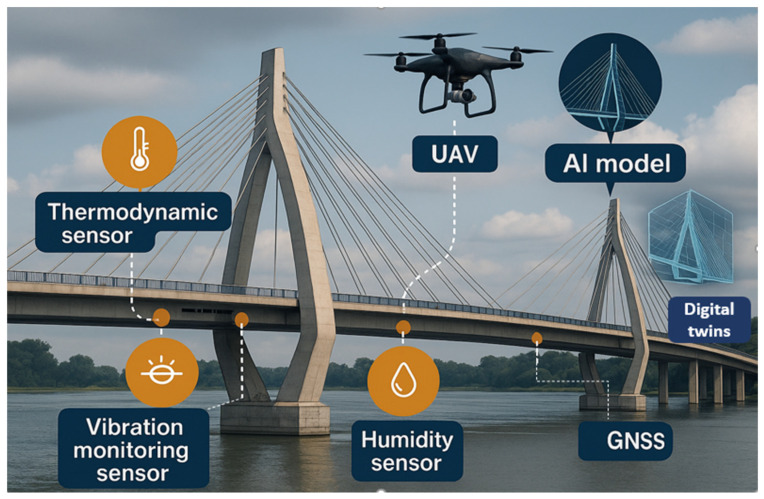
Advanced technologies used on bridges.

In Figure 4, all the advanced technologies available for SHM are presented. The orange bubbles serve as sensors for the IoT-based SHM, monitoring vibrations in both vertical and horizontal directions. The other sensor measures the temperature of the bridge and of the road. Likewise, drones scan the entire bridge, generating a point cloud that is used to build a digital twin. Lastly, an applied AI model can detect cracks, corrosion, and other defects to inform about the current structural condition of the bridge.

### 2.1. IoT, Computing, and SHM Systems

IoT systems aim to monitor and manage physical quantities related to the “things” to which they are applied. Thus, the measurement of these physical quantities plays a crucial role [11]. In the monitoring process, technologies of IoT, Computing, and SHM systems play a crucial role in data acquisition. This data comes from a wide range of variables such as humidity, temperature, load, vibration, and even position. Based on the data obtained, it is possible to predict possible failures on a bridge or other infrastructure object. Subsequently, the next step in the monitoring process, which is visual inspection, can confirm these failures, although they are not always easily visible.

In the context of SHM, modern systems utilize IoT sensor platforms with so-called edge processing for local data analysis, as demonstrated in studies on the Forth Road Bridge using the GeoSHM system. This approach enables quick decision-making but also introduces requirements for data reliability and cybersecurity [12]. SHM systems integrate a wide range of sensors (accelerometers, strain gauges, optical fibers, humidity sensors) that monitor dynamic and environmental parameters in real time. An example is the Illinois Structural Health Monitoring Project (ISHMP), which implemented wireless sensors on the Jindo Bridge in South Korea, thereby reducing the cost of installing and maintaining data networks [13]. The article by Aktan summarizes decades of experience with SHM on bridges and emphasizes the role of cyber-physical systems, including the challenges related to analyzing and interpreting large data streams [14]. Their research supports interdisciplinarity among civil engineering, IT, and infrastructure. A software tool that enables such integration is Building Information Modeling (BIM). BIM enables multiple professionals to work on a single project file by editing and contributing their respective parts while viewing the modifications and contributions of other collaborators in real time.

Thanks to technological advances in the Internet of Things, including sensors, communications, and informatics, real-time monitoring of long-span bridge structures has become increasingly practical and reliable. Many bridges have implemented SHM systems to reduce the potential risks of service outages or damage worldwide. The entire SHM system generally consists of a sensor module, a data collection and transmission module, a data processing and management module, and an evaluation and reporting module. Accordingly, many researchers have conducted studies on this topic, focusing particularly on damage identification, the application of global navigation satellite system (GNSS) sensors, extreme event analysis, and SHM systems. GNSS technology has made significant progress in recent decades, making GNSS positioning a reliable sensor for monitoring deformations of medium- and long-span bridges [15]. To improve positioning accuracy, the influence of GPS satellite geometry and pseudolites on structural deformation monitoring has been analyzed. Integrated GPS and triaxial accelerometers were deployed to detect dynamic response. Modal frequencies of a suspended medium-span bridge were obtained using multi-mode GNSS positioning and multipath mitigation. A method for extracting GNSS displacement components for long-span bridges and verifying it using SHM data were proposed in [14,16,17].

### 2.2. UAVs/Drones and Computer Vision

Unmanned aerial vehicles are primarily used for monitoring infrastructure due to their ability to access locations that are difficult or, in some cases, entirely inaccessible to humans. In some instances, this does not involve drones in the strict sense, but rather robots capable of overcoming various obstacles. As a result, inspectors are not exposed to the risks associated with inspecting infrastructure assets [16]. Drones enable non-contact visual inspection of hard-to-reach areas of bridges. When combined with artificial intelligence models, they are used to detect cracks, corrosion, or destructive changes. For example, in the study by Zhang et al. [17], a UAV system with crack mapping integrated into GIS is proposed, achieving a damage identification accuracy rate of 80.5%. Another study presents a method based on the YOLOv7-CD approach for fast and efficient real-time crack detection on bridge structures [18]. Within NATOs science and security programs, several conferences have been devoted to this issue. The book focused on the use of drones in the monitoring and protection of critical infrastructure by unmanned systems. Also refer to this topic [19,20].

From a state-of-the-art perspective, UAV-based bridge inspection has evolved from purely visual documentation toward more structured workflows that aim to produce consistent, traceable, and quantifiable outputs. Review work on UAS bridge inspection highlights typical advantages (rapid coverage, reduced need for access equipment and traffic disruption, improved safety) while also stressing recurring limitations: dependence on lighting and weather, sensitivity to flight stability, line-of-sight constraints, and challenges in capturing fine defects at sufficient image resolution [21,22]. A prominent direction is to move beyond images as evidence toward images as measurement, using photogrammetry or image-based 3D reconstruction to create a permanent digital record that can support off-site inspection and repeated assessments. Chen et al. show that 3D reconstruction quality and geometric measurement accuracy depend strongly on data-collection choices (e.g., path-planning and ground sampling distance), implying that inspection-grade results require deliberate acquisition planning [15,23]. This emphasis on acquisition design is also evident in reviews of deep learning for concrete-defect detection: Zhang et al. note that UAV acquisition is efficient and safer for operators but can suffer from signal reception issues in complex terrain or indoor settings, as well as from image overlap and irregularity. The most significant step change in UAV/robot visual inspection is achieved when imagery is paired with artificial intelligence (AI), particularly deep learning, to convert raw data into actionable condition information (e.g., crack localization, spalling segmentation, corrosion detection, and multi-defect mapping). A comprehensive review of computer-vision methods for bridge inspection summarizes the typical pipeline (data acquisition, defect recognition, measurement, and reporting) and discusses how platforms such as UAVs and robots enable large-scale surface-defect monitoring when combined with modern learning-based computer vision [24]. In learning tasks, recent literature commonly distinguishes among classification, object detection (bounding boxes), and semantic segmentation (pixel-level defect delineation), with segmentation being particularly valuable for quantifying crack geometry or spalled areas [17,24]. For bridge-specific crack detection, Yang et al. propose a real-time approach that integrates UAV image acquisition with an improved target detection algorithm and transfer learning to enhance practical deployability in field conditions [19]. Beyond cracks, UAV imagery is also used to detect broader defect categories; for instance, improved Mask R-CNN variants have been reported to identify multiple defect types in UAV-collected images of reinforced concrete bridges and to generalize better than baseline networks [25].

### 2.3. Artificial Intelligence

Systems based on deep neural networks, also known as CNNs (Convolutional Neural Networks), enable the automatic extraction of features from image data for classification, crack detection, semantic segmentation, and other applications. Comprehensive overviews of this topic include studies by Hamishebahar [26] and a 2025 review published in Scopus. The latest methods include architectures such as ConvNext, combined with encoder-decoder and dual-channel CNNs, which achieve up to 92% accuracy in real-world UAV imagery scenarios [27].

Artificial intelligence is particularly useful in bridge monitoring by transforming dispersed images and sensor data into consistent, actionable information. From aerial and ground imagery (UAVs, cameras), it can automatically detect and quantify defects, like cracks, spalling, corrosion, or leaking joints, and, through semantic segmentation, distinguish a true defect from a shadow or dirt. Thus, from the perspective of the monitoring process, AI models are most commonly used in intelligent diagnosis. By comparing time series, it highlights changes between repeat inspections and simultaneously oversees photogrammetric quality and image coverage so that hard-to-access areas are not missed. In sensor data (strain, vibration, temperature, humidity, and GNSS), it recognizes anomalies and drift, refines operational modal analysis, and, through multisource fusion, builds robust structural health indices. This shortens the time to detect early damage and supports predictive maintenance with less disruption to traffic [18].

AI also accelerates decision-making. It translates output into risk scores at both the element and bridge level, emphasizes uncertainty (confidence) for each finding, and, in a human-in-the-loop mode, allows the inspector to quickly confirm or correct the proposed action. Explainable models (XAI) improve auditability and trust, while integration with the digital twin and asset-management tools (BIM/CMMS) automatically generates work orders, suggests intervention timing, and simulates what-if scenarios. Even at the mission-planning stage, AI optimizes UAV flight paths to bridge geometry and airspace constraints, and during operations it safeguards data quality, model calibration, and security aspects (e.g., suspicious telemetry patterns or spoofing attempts). The result is fewer blind spots between periodic inspections, lower detection latency, more consistent assessments, more targeted interventions, and measurable savings while maintaining, or often increasing, safety levels [27,28].

When training an artificial intelligence model for data classification, significant time, training data, and, depending on the type, a human specialist are required to program the model effectively. This is because, during preprocessing, parts of the image that the AI model has not yet recognized must be manually adjusted for recognition [28].

### 2.4. Digital Twin

**Figure 5:**
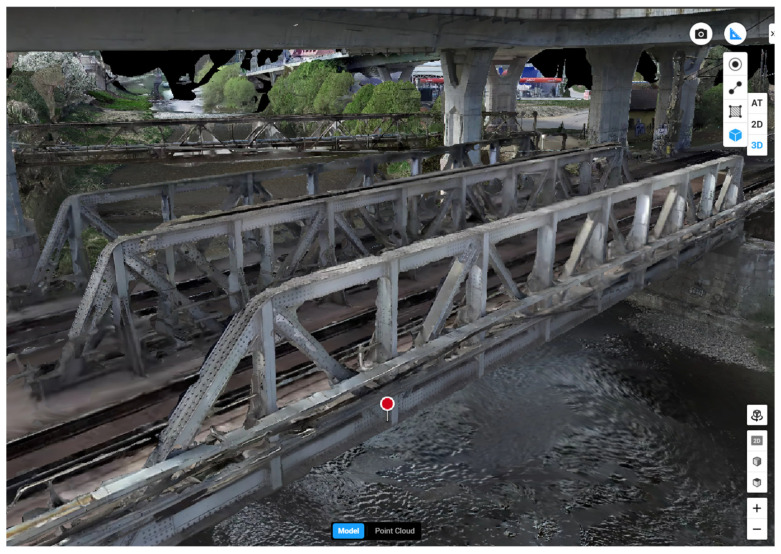
Visualized point cloud of the steel railway bridge in Čadca.

The Digital Twin (DT) is a technology that is currently very popular, especially in Asian countries such as China, Singapore, and South Korea. A DT is an exact virtual replica of a real-world object, created by capturing a point cloud (Figure 5). The point cloud can be generated using high-quality scanning devices or total stations. This technology, in combination with sensors that monitor precise position, temperature, and other parameters, can provide real-time information about the object’s external condition and, depending on the sensors used, its internal condition as well [27]. DTs are currently widely used not only for monitoring critical infrastructure but also for manufacturing processes, visualizing specific point elements, and predictive design. In various projects, DTs have been applied, for instance, in the NATO IRIS project, the DESDEMONA project, the APRIORI project, and in the phase of realization of the Slovak Republic in the REMAKE 3D project [28,29,30,31].

In state-of-the-art bridge DT frameworks, the integration logic typically follows a monitoring chain: data acquisition from specific sensors and inspection data, data management and quality control, model updating or data assimilation (e.g., calibrating FE models), and then decision support (e.g., maintenance planning, prioritization, and what-if analyses [32]). Recent bridge maintenance-focused reviews emphasize that in the monitoring process, DTs can support inspection planning, predictive maintenance, and resilience assessment, but they also underline persistent challenges: unclear or inconsistent DT definitions in practice, lack of standardized data schemas and interfaces, uncertainty quantification, and the effort required to maintain an up-to-date model over time [33]. At the implementation level, bridging the gap between BIM/BrIM and “engineering-grade” structural analysis is a recurring theme, motivating workflows that enable bidirectional mapping between geometric information models and numerical simulations [34,35].

From the perspective of creating the “virtual replica,” the geometric backbone of DTs is commonly derived from reality capture, where a point cloud serves as an input for reconstructing an as-is model. In practice, point clouds are generated using terrestrial laser scanning, mobile mapping, photogrammetry, or total stations; the main objective is to obtain sufficiently accurate geometry and to enrich it with semantic information such as elements, materials, and component IDs so that inspection results and sensor measurements can be linked to specific bridge parts [32,33]. Recent bridge-focused DT literature highlights that the scan-to-BIM step is often a bottleneck. Point clouds are imperfect (noise, incomplete coverage), and converting them into parametric models suitable for simulations and asset management still requires robust automated or semi-automated methods. This is a key reason why the notion of an “exact virtual replica” is increasingly replaced by a more realistic view: DTs operate with multi-fidelity models, where geometric detail is highest where it supports inspection and assessment tasks, while other parts may remain simplified to maintain computational efficiency and updateability [34,35].

When implementing modern technologies, the focus is primarily on improving safety, whether through advanced automated analyses, better visualization, or enhanced output data. However, the risks associated with the implementation and operation of modern technologies are often overlooked. The adoption of modern technologies, particularly in infrastructure applications such as bridge monitoring, brings numerous benefits but also several risks that can be categorized into five groups based on their origin. The identified risks fall into the following categories: technical, cyber, legislative, economic, and organizational. These risks arise from the following SWOT analysis.

## 3. SWOT Analysis

The reason for applying a SWOT analysis is its ability to comprehensively identify and evaluate the key factors influencing the implementation of modern technologies for the monitoring of (critical) infrastructure. It enables the highlighting of not only the significant advantages of these technologies, such as increased accuracy, faster response times, and process automation, but also internal limitations that may reduce their effectiveness, such as the need for specialized personnel or high initial costs. At the same time, the SWOT analysis reveals opportunities to develop innovative approaches, as well as threats (risks) arising from implementing these technologies, particularly in cybersecurity, legislative uncertainties, and ethical concerns related to data collection and processing. In the SWOT analysis the following aspects were identified:

**Strengths of advanced technologies**

*High accuracy in data collection and evaluation* is the main reason why technologies such as SHM systems, IoT, and DTs are implemented. Probability of detection (POD) is an established metric for evaluating many investigations aimed at detecting a specific property of a subject of interest. For instance, it has many applications in Non-Destructive Evaluation for identifying defects within structural architectures. It can easily be used in SHM systems, serving as a compact, more integrated evolution of the former technology. Fiber Bragg Grating and other sensors (strain, vibration) offer high sensitivity, long-term stability, and resistance to interference, making them suitable for bridges [36,37].

*Real-time monitoring of structural conditions* is significant for ensuring the safe operation of infrastructure systems, despite the high costs of traditional methods. Real-time monitoring is provided by SHM systems (e.g., temperature and vibration sensors); otherwise, it can be carried out frequently and regularly using drones or robots with the necessary equipment. The paper of Sun, et al. [38] presents the development of a real-time DT of a laboratory-scale bridge to assist in infrastructure monitoring. Traditional methods of visual inspection and non-destructive tests are generally undertaken to monitor and evaluate the structural health of the infrastructure. However, these methods lack reliability due to the need for instrumentation calibration and reliance on subjective visual judgments. For example, technology as a DT digitally replicates existing infrastructure assets (bridges, railways, buildings, etc.), offering significant potential for real-time intelligent monitoring and assessment of structural health [39].

*Support for predictive maintenance and failure prevention* is provided by integrated SHM systems and DTs, which use mechanism analysis, monitoring technology, and data analytics to diagnose, classify, locate, and assess the significance of structural conditions (e.g., sudden or cumulative damage) to ensure the functionality and operation of bridges. Integrated SHM systems have improved the maintenance, management, and decision-making of bridges by continuously monitoring and evaluating working conditions [40,41]. 

*Integration with intelligent infrastructure (smart cities)* lies in SHM systems and the use of DTs. As mentioned, DTs can significantly alter how cities are managed and planned, as exemplified by cities like Singapore and Dubai. Peldon points out the main hurdles, like gathering data, connecting systems, handling vast amounts of information, and making different technologies work together [42]. The research synthesis highlights the dynamic nature of DTs, their multifaceted technological layers, and their instrumental role in shaping sustainable urban futures. Despite the promising outlook, the study also highlights several technological and real-world hurdles that must be addressed to fully unlock DTs’ capabilities in urban environments [43]. Integration of smart cities is spreading from Houston to Singapore, and managers around the world are using sophisticated digital tools (such as DTs) to monitor and control aspects such as groundwater, manage urban heat islands, reduce air pollution, and effectively manage their waste [41].

**Weaknesses of advanced technologies**

*High initial and operational costs* associated with an SHM solution can be challenging for prospective buyers or sellers to comprehend. Some hidden costs can sometimes be challenging to estimate a priori. Inaudi [44] defined a list that can help in establishing the main costs that are usually associated with the implementation of an SHM system: Immediate costs/capital investments SHM design costs, including integration with the structure’s design, Hardware costs (sensors, cables, data acquisition, data management hardware, communication hardware), Installation costs, including integration with the building schedule, configuration and commissioning, Costs for installation reporting, as-built documentation, and system manuals Operational costs: System maintenance, spare parts, consumables, energy, communication costs, Data management costs, Data analysis, interpretation, and reporting costs [45]. For clearer interpretation, Table 1 summarizes the technologies used in bridge monitoring, their principal limitations, and indicative cost ranges.

Conventional monitoring systems—based on wired sensors; GNSS; or geodetic systems—often require costly installations and intrusive interventions and offer limited spatial or temporal resolution. As a result, the capacity to detect early signs of structural deterioration remains inadequate, particularly in regions where maintenance resources are scarce or where infrastructures operate under harsh environmental and dynamic conditions. Recent advances in artificial intelligence, computer vision, and photogrammetry have opened new possibilities for contactless, scalable, and cost-effective monitoring technologies.

Another identified weakness is the *Need for qualified personnel and IT infrastructure*. The operation of advanced SHM/IoT monitoring systems is not only a technical or hardware challenge but also depends on qualified personnel who can correctly install sensors, maintain them, manage calibration, interpret complex multi-source data, and translate that information into maintenance and safety decisions. In practice, many infrastructure operators face a skills gap because these systems require combined expertise in structural engineering, data analysis, network/communication technologies, and cybersecurity, a profile that remains relatively rare. At the same time, modern monitoring requires a robust IT infrastructure: reliable edge devices, secure connectivity, protected data storage (often cloud-based), real-time analytics pipelines, firmware version control, and controlled access management. Without both skilled staff and a stable IT/OT environment, the risk of false alarms, missed warnings, downtime, or misinformed decisions increases sharply [45,46,47].

Discussing sensors and the probability of detection, the next weakness is the *Possibility of malfunctions and false alarms*. The successful implementation of SHM systems is constrained by the ability to evaluate their performance, reliability, and durability. Although many SHM techniques can detect, locate, and quantify damage in various types of structures, their certification processes remain limited. Despite the efforts of academia and industry to define methodologies for the performance assessment of such systems in recent years, many challenges remain unsolved, such as false alarms that can occur under high load or high sensitivity [36]. False alarms can be caused by environmental factors, such as temperature fluctuations, humidity variations, seismic activity, and other external factors, which can significantly affect the performance of SHM equipment. These changes can impact the accuracy and reliability of the collected data, potentially resulting in false alarms or missing issues. Furthermore, the aging process poses a fundamental challenge for SHM systems. Over time, the components of these systems can undergo wear and tear, resulting in reduced functionality and sensitivity [48,49]. The feasibility of operational digital twins at a network scale is strongly linked to affordable sensing and data-transfer architectures; low-cost wireless SHM concepts for bridges are therefore a key enabler for continuous updating of DT states [50].

**Figure 6:**
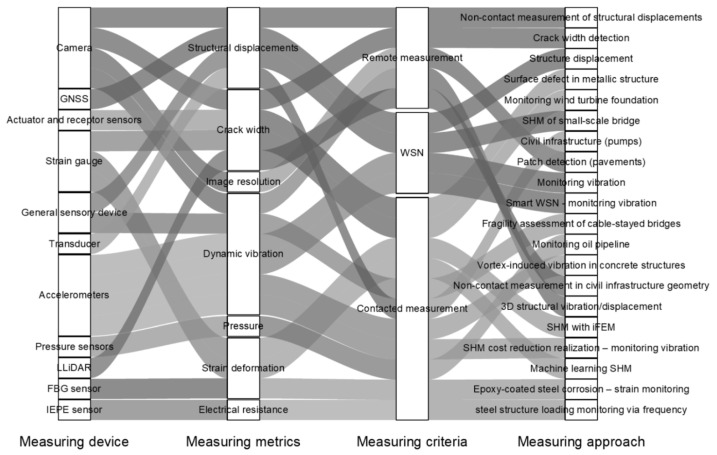
Data acquisition methods for civil infrastructure [38].

Lastly, *dependence on specific technology providers* can also be a serious obstacle for potential new adopters of advanced monitoring technologies. Vendor lock-in can limit flexibility, increase long-term costs, and make it challenging to integrate new components or switch suppliers without losing functionality [41]. This is especially relevant in complex SHM/IoT ecosystems where hardware, software, data platforms, analytics, and visualization tools are tightly coupled and often proprietary. As illustrated in Figure 6 (Data acquisition methods for civil infrastructure), even a simplified operational flow may require at least 11 distinct devices or subsystems, including sensors, edge gateways, communication modules, storage, analytics engines, and visualization interfaces, such as LiDAR. If these components are sourced from different vendors with incompatible standards, integration becomes expensive and fragile; if they are all sourced from a single vendor, the customer becomes dependent on that vendor’s pricing, service, update policy, and technical roadmap [38,51].

Figure 6 also provides an overview of the different types of sensors used in the latest advances in DT and the specific metrics that different researchers in the field of civil infrastructure have already measured [52,53,54,55]. Also, Figure 6 underlines how many different sensors are implemented nowadays for the data flow of DT. Moreover, Figure 6 highlights the increasing multi-sensor character of contemporary DT dataflows, where several sensor families are implemented simultaneously to improve observability, redundancy, and diagnostic confidence. This is important because different sensor types operate on different spatial and temporal scales: high-rate response sensors capture short-term events and dynamic behavior, while geometric and inspection sensors support periodic updates of the as-is state, and environmental sensors enable normalization (e.g., temperature compensation) and contextual interpretation. Consequently, modern DT implementations often require sensor synchronization and metadata management, data quality control and validity tagging, and fusion of heterogeneous outputs into unified DT state variables. In this way, the figure visually underlines that DT effectiveness depends not only on the presence of a virtual model but also on the breadth of sensing and the robustness of the end-to-end data pipeline that feeds and updates the twin [52,53,54,55].

**Opportunities for using advanced technologies**

There are numerous opportunities to discuss the implementation of advanced technologies. First, the *Development of smart infrastructure and digital twins* can provide valuable data for decision-making processes. Real-time SHM using edge AI, uncertainty quantification, and transfer learning enhances adaptability across diverse structures. Three-dimensional reconstruction and digital twins facilitate virtual inspections and predictive maintenance. These AI methodologies enhance precision, efficacy, and decision-making in SHM applications. Integrating AI into SHM enhances the reliability of concrete bridge assessments through automated inspections, refined data analysis, and the facilitation of predictive maintenance programs [41].

*Funding opportunities for research and innovation* are almost limitless nowadays. Many international grants focus on innovation and the development of digital infrastructure, including smart cities, smart infrastructure, and the implementation of AI models to enhance infrastructure management. Several international projects aimed at enhancing critical infrastructure through the implementation of advanced technologies were carried out in recent years, including Project IRIS and Project APRIORI, sponsored by NATO SPS; Project DESDEMONA; and Project REMAKE 3D, the main results are presented here [28,29,30,31].

The next identified opportunity is *Improved crisis response and preventive measures*. Advanced monitoring architectures that combine sensor networks, real-time analytics, and DTs can significantly enhance both crisis response and preventative maintenance. By continuously measuring structural behavior (e.g., strains, deformations, load effects) and feeding these data into a calibrated analytical or numerical model, the system can rapidly identify whether observed damage is local or critical to overall stability [17,34]. This allows infrastructure managers to make faster decisions during emergencies, such as after an impact or overload, rather than waiting for manual inspection. At the same time, DT systems serve as long-term diagnostic tools, tracking the progression of fatigue, corrosion, and material degradation rather than only during scheduled inspections. That supports proper predictive maintenance, because interventions can be planned before a defect escalates into a failure. These approaches also reduce dependence on subjective human visual assessment in hard-to-access areas, which is vital for aging bridges. Overall, the integration of sensing, intelligent processing, and digital representation enables infrastructure to be managed not only reactively after an incident but also proactively to avoid one [37,51].

In the end, *International standardization and cooperation* are among the most significant opportunities because they enable the sharing of ideas, skills, and experiences worldwide. An example of how know-how from the United Kingdom has been transferred to Asian bridges. It deals not only with sensor and GNSS technology but also with data management strategy, long-term deformation analysis, and links to bridge operational safety. In other words, it is not just about measurement but also about standard processes and data interpretation [15,56].

**Threats of advanced technologies**

Speaking of digital space, undoubtedly, the most crucial are *Cyberattacks and data manipulation*. While the benefits of implementing a structural health monitoring system are attractive, there are growing concerns that integrating technology into modern infrastructure systems, such as bridges, dams, power stations, and other infrastructure assets, can make these systems vulnerable to cyberattacks [57]. For example, a cybercriminal or national stakeholder could be interested in exploiting information collected by sensors to assess a bridge’s condition and transmit the results. Moreover, there are concerns that malicious actors could also inject false information about the bridge’s condition, triggering an alarm for the bridge operator. The reality is that, should any of these cyberattacks succeed, the attack could have broader implications for both the cyber and physical aspects of the infrastructure (bridge) under attack [58].

One of the subsequent risks linked to the use of UAVs and IoT is *Legal and ethical issues*. Privacy by design is essential because the General Data Protection Regulation (GDPR) requires organizations that process personal data to apply the principles of data protection by design and by default (e.g., drone pilots and operators). These principles aim to ensure that data protection and privacy considerations are incorporated into the design and development of new technologies, processes, and flight plans from the very beginning, and that as little personal data as possible is processed by default [51,59,60].

*The rapid obsolescence of technology* could be threatening, as well as the other two threats mentioned above. Obsolescence poses several technological risks that can significantly impact an organization’s operations, security, and financial health and is closely related to cyberattacks and delayed or inaccurate data flows [61]:

- Increased vulnerability to cyberattacks: Outdated systems often lack the necessary security features and updates to protect against modern cyber threats, leaving organizations susceptible to data breaches and malware infections.
- Operational inefficiencies: older technology can hinder productivity due to slower performance, more frequent breakdowns, and incompatibilities with newer systems or software.
- Compliance risks: Many industries have regulations that require up-to-date security measures. Using obsolete technology can lead to non-compliance, resulting in fines and legal penalties.
- Reputation damage: Failures or security breaches caused by outdated technology can damage an organization’s reputation, eroding customer trust and competitive standing.

A fourth identified threat that may be relevant, at least in some countries, is *Insufficient regulation or unclear responsibilities*. In the Slovak context as a case study, several advanced monitoring technologies, most notably UAV-based bridge inspections, permanent IoT/SHM sensing, AI-based damage detection, and digital twins, are considered via scientific projects. The first case studies, however, are not yet embedded in binding operational procedures of bridge monitoring for infrastructure operators. The legal framework relies mainly on general European regulations (for example, EASA [59] rules for drones and GDPR for data handling). Still, there is no unified national methodology that would clearly define who is allowed to use these technologies, who owns and manages the collected data, who is responsible for interpreting the data, and who has the authority to make operational decisions (such as traffic restrictions or emergency maintenance) if sensors or an AI model indicate a risk. In practice, this can lead to situations in which the technology provider, the bridge owner/operator, and the state authority have different expectations, such as when a sensor reports an anomaly. Still, it is unclear whether this should trigger an immediate traffic limitation, an urgent visual inspection, or simply an entry into the archive. In addition, for smaller bridges managed by regions or municipalities, it is often not defined who is even capable of operating such a system in the long term (sensor maintenance, calibration, cybersecurity). This uncertainty creates a risk of delayed reaction, shifting responsibility between stakeholders after an incident, and strategic investment postponement (“the technology is installed, so it must be safe”) without a clearly defined duty to act on the data. In other words, without a clear governance framework, Slovakia can formally adopt advanced monitoring technologies but remain vulnerable when a critical decision needs to be made in a crisis [62,63].

**Figure 7:**
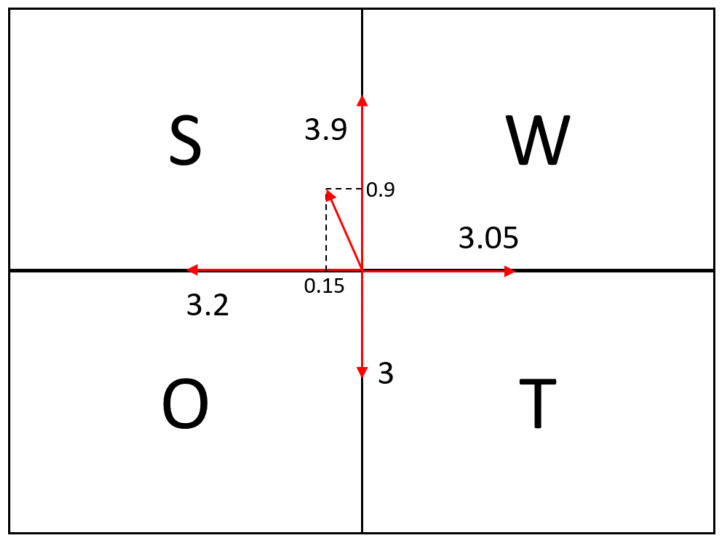
Weighted SWOT Diagram of risk analysis.

When implementing modern technologies, it is crucial to prioritize enhancing the safety and resilience of critical infrastructure assets. On the other hand, it is necessary to consider the threats posed by modern technologies as they are in Table 2. And the following Figure 7 points out a strategy that can be implemented. This means that advanced technologies now have the best opportunity to grow and help enhance the resilience of bridges worldwide.

The results of the SWOT analysis (Figure 7) point toward an offensive (growth-oriented) strategy, indicating that advanced monitoring technologies have substantial potential for broader adoption and scaling within bridge management. In particular, the identified strengths and opportunities suggest that these technologies can improve the accessibility of inspections, increase the objectivity and comparability of condition assessments, and enable earlier detection of degradation through continuous or near-continuous data streams. At the same time, the analysis makes clear that the risks and threats associated with modern technology deployment are non-negligible and must be addressed systematically. Key concerns include data quality and reliability issues (e.g., sensor deviation, missing data, or insufficient image quality), uncertainty and bias in AI-based interpretation, cybersecurity and privacy constraints linked to IoT connectivity and UAV data acquisition, and organizational challenges such as skills, governance, and long-term maintenance of monitoring systems. For better understanding, they will be identified and examined in the next chapter.

## 4. Risks Associated with the Implementation of Advanced Technologies

For security and crisis managers, advanced technologies represent key decision-making tools for rapid, sophisticated analysis while simultaneously posing additional risks that must be managed. However, it can be concluded that modern technologies represent a greater opportunity for society than threats. In Table 3, risks are displayed for the use of advanced technologies for monitoring bridges and categorized into four categories: Technical, Cybersecurity, Legal and Ethical, and Organizational and Operational. The table provides the value of each risk calculated as

Risk (R) = Probability of the event (P) × Impact of the event (I)(1)

Risk values were derived via expert elicitation. Each risk was rated for probability and impact on a 1–5 scale by 5 experts with backgrounds in bridge inspection, SHM, and asset management. Ratings were aggregated using the median to reduce sensitivity to outliers. Disagreement was assessed; the second discussion round was then conducted, and ratings were repeated. The final risk score was computed as Probability × Impact. The implementation of modern technologies in bridge monitoring increases accuracy and efficiency but also requires systematic risk management. The best approach lies in the combination of

- traditional and digital methods,
- system backups,
- personnel training,
- properly established legislation and cybersecurity policies.

### 4.1. Technical Risks of SHM Systems

Hardware or software failures of sensors, power outages, incorrect calibrations, or inadequate data management can lead to false alarms or missed detection of critical states. Aktan et al. highlight the challenges related to synchronizing and archiving large data volumes [14]. Key metrics and reliability concepts are closely related to the probability of defect detection. That is, whether it is possible to detect a fault using the applied sensors, but of course, if it is possible to detect a sensor losing its reliability. This is a key metric used to assess the likelihood that a system will detect hidden structural damage. The probability of detection is especially relevant for techniques such as ultrasound and guided waves. Reliability studies also consider the system’s ability to localize the defect and estimate its size accurately [64].

Information quality metrics are new metrics that evaluate the impact of data quality on the reliability of structural models. They include factors such as sensor precision, signal resolution, and the effect of environmental noise on data interpretation. Bayesian approaches are often used to update reliability assessments in real time as new data are collected [65].

**Figure 8:**
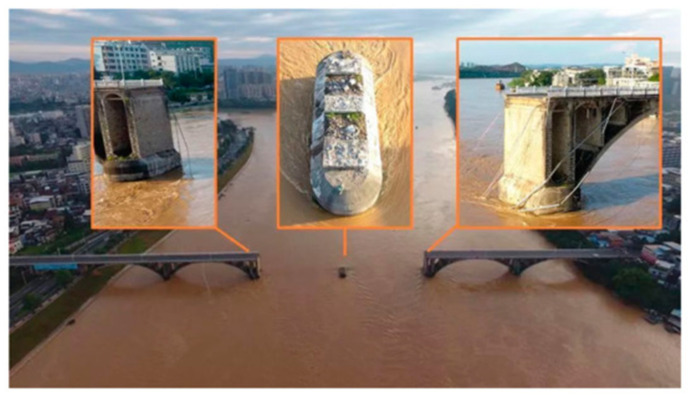
Collapsed bridge of Zijin due to inactive sensors [68].

These days, it is not typical for bridge collapses to stem from sensor malfunctions, unreliable measurements, or fake data, because the primary reason for bridge collapse is structural conditions. In Singapore, it was found that strain-detecting sensors installed on the retaining wall of an excavation site were buried 2 days before the collapse, and no readings were taken. Despite recorded movements, supervisors failed to act on the data. Cracks were treated superficially, and no corrective measures were undertaken [66]. On the other hand, there are many cases where sensors were inactive due to roadworks, with immediate consequences, such as the Morandi bridge in Italy and the Zijin bridge in China (Figure 8). These extraordinary events could be prevented by other external systems (regular image processing, UAV monitoring, etc.) because implemented sensors must be inactive [67,68].

New methodologies are emerging that assess SHM reliability using quantitative indicators such as PoD, defect localization, and sizing performance. Sensor network layout and measurement error propagation are also considered [69,70].

### 4.2. Cybersecurity

All connected systems are vulnerable to cybersecurity threats. Cybersecurity threats for IoT systems are commonly classified according to the CIA triad: Confidentiality, Integrity, and Availability [67]:

- Confidentiality: Ensures that sensitive SHM data (e.g., structural conditions, operational parameters) is accessible only to authorized users and systems. Breaches can lead to privacy violations, competitive intelligence leaks, or targeted attacks on critical infrastructure.
- Integrity: Guarantees the accuracy, authenticity, and trustworthiness of SHM data throughout its lifecycle. Compromised integrity can result in false alarms, missed detections, or inappropriate maintenance actions, potentially endangering public safety.
- Availability: Ensures that SHM services and data are accessible when needed. Attacks on availability (e.g., denial-of-service) can disrupt monitoring, delay emergency responses, and undermine trust in the system.

Most common attacks targeting one or more of the three above-mentioned aspects include:

- Denial-of-Service attacks: consisting of the attacker flooding the network or specific devices with excessive traffic, overwhelming their processing or communication capabilities, and making the system unreliable for legitimate monitoring and control; in some cases, the attack may be aimed at draining the battery or power resources of wireless sensor nodes, causing premature device failure and persistent monitoring gaps [59]. Such attacks compromise the availability of the SHM system.
- Eavesdropping attacks: involving passive monitoring of network traffic to extract sensitive information. Such attacks can be easily conducted on unencrypted or barely protected wireless transmissions, such as Wi-Fi or LoRa. Eavesdropping attacks compromise the confidentiality of SHM data.
- Man-in-the-Middle (MitM) attacks: occurring when an adversary intercepts, alters, or relays communications between SHM devices and backend systems without detection. Such attacks can be conducted at the network level by exploiting ARP (Address Resolution Protocol) spoofing or vulnerabilities of higher-layer protocols, such as MQTT (Message Queueing Telemetry Transport) without TLS (Transport Layer Security). MitM attacks compromise both confidentiality and integrity.
- Malware and botnet: consisting of the injection of malicious code into an IoT system once access is gained to the network. Attackers exploit weak credentials, unpatched firmware, or default configurations to compromise devices. Malware can disrupt SHM monitoring, exfiltrate sensitive data, and transform SHM devices into launchpads for further attacks. Malware can compromise all three aspects of availability, integrity, and confidentiality.

The actual risk associated with the cybersecurity of an SHM system should be carefully evaluated depending on the nature and the maintenance level of the exposed devices and network, the used protocols, and their security.

### 4.3. Legislative and Ethical Risks

The use of UAVs, IoT, and cloud computing raises questions about GDPR, personal privacy, and liability in the event of sensor or AI model failure. National-level standards are only partially developed. In the future, it is necessary to improve this field through strategic management, such as what the European directive is, which should be binding across the entire European Union, as there are differences between countries. The growing interest in UAVs for commercial and recreational purposes requires clear rules for their safe use. The European Union entrusted this task to the European Union Aviation Safety Agency (EASA) [59], which was tasked with designing and harmonizing drone regulations across the EU.

As a result, Regulations of EU 2019/945 [71] and 2019/947 [72,73] were established, which define the conditions for registering drone users, categorize UAS (Unmanned Aerial Systems), and outline rules for safe operation. These regulations have been in effect since 31 December 2020, but their application varies across individual EU member states. The European Union has introduced rules for the use of drones, classifying unmanned systems into three categories: Open, Specific, and Certified. The Civil Aviation Authority is responsible for managing this classification. Table 4 provides an overview of identified risks by impact area (privacy, AI models, safety, social impacts, and abuse). The examples listed represent the most common risk mechanisms that may arise when deploying UAVs, SHM based on IoT, AI, and DT in bridge infrastructure.

Ethical risks associated with the use of advanced technologies in infrastructure monitoring primarily concern privacy, transparency, accountability, and the broader social impact of these systems. One of the most pressing concerns is the unauthorized collection of sensitive personal or location-related data, particularly using UAVs and smart sensors. This raises questions about surveillance, consent, and the boundaries of personal privacy.

In the case of artificial intelligence and machine learning models, ethical concerns arise from potential bias in the data used for training and a lack of transparency in decision-making processes. These models may provide assessments or recommendations that significantly influence infrastructure management, yet their decision-making processes often remain a “black box,” undermining public trust. The European Union regulates artificial intelligence through the AI Act to protect the safety, privacy, and fundamental rights of citizens while maintaining confidence in digital services [70]. The regulation adopts a risk-based approach: the higher the risk of an AI use case (e.g., in critical infrastructure, healthcare, or rights-impacting decisions), the stricter the requirements for data quality, risk management, human oversight, and cybersecurity. It also establishes transparency and accountability rules to ensure that systems are explainable, auditable, and traceable throughout their entire lifecycle. A common framework prevents regulatory fragmentation across member states and strengthens the single market, facilitating the cross-border deployment of trustworthy AI. Innovation mechanisms such as regulatory sandboxes allow new solutions to be tested without compromising safety and rights. Finally, post-market surveillance and sanctions ensure that the rules are not only declared but also enforceable, preventing harm and misuse [71]. There are also risks related to emotional trust and public perception. Citizens may feel discomfort or distrust toward technologies that constantly monitor public spaces, especially if they are not correctly informed about the purpose, scope, and data handling of these systems. This can lead to resistance or rejection of even beneficial innovations.

Another serious ethical issue concerns the potential misuse of technologies, such as drones, being used not only for monitoring but also for surveillance or even violent purposes. A notable example is the public controversy surrounding the U.S. company Axon, which considered equipping drones with weapons for public safety, leading to backlash from ethics committees.

Lastly, in terms of social impacts, technological implementation may result in job displacement, especially among inspectors and maintenance personnel. Furthermore, access to and benefits from such advanced systems may not be equally distributed across regions or institutions, raising concerns about equity and justice. The lack of comprehensive ethical and regulatory frameworks presents a risk. Many advanced technologies are deployed faster than legislation can adapt, creating a regulatory vacuum. Therefore, it is crucial to develop guidelines and ethical design principles that ensure these technologies are implemented in a manner that respects human rights and advances the social good.

### 4.4. Organizational and Operational Risks

Unclear governance and decision-making during anomalies are among the most significant risks. One case occurred in Miami in 2018. The National Transportation Safety Board (NTSB) identified design errors and insufficient peer review as the probable cause of the bridge collapse. It also noted that, despite pronounced cracks, the roadway was not closed, and work continued, constituting a failure of decision-making procedures and risk management. The 174-foot-long (53 m) bridge span fell about 18.5 feet (5.6 m) onto SW 8th Street, which consists of four travel lanes and one left-turn lane in the eastbound direction and three travel lanes in the westbound direction. Two of the westbound lanes below the north end of the bridge were closed at the time of the collapse; however, one westbound lane and all five eastbound lanes remained open. On the day of the collapse, a construction crew was working on retensioning the post-tensioning rods within member 11, which connected the bridge canopy to the deck at the north end. Eight vehicles located below the bridge were fully or partially crushed. One bridge worker and five vehicle occupants died. Five bridge workers and five other people were injured [51].

Secondly, there is a risk of ignoring or not acting on measurements and alarms, like alert fatigue and missing SOP. The real-life case of Nicoll Highway in Singapore, in 2004, has already been mentioned. Instrumentation in some locations was buried, readings were not taken for 2 days before the collapse, and warning signs were not adequately addressed, a typical organizational failure in handling data and alarms. And of course, the human factor cannot be forgotten. The next risk mentioned is insufficient qualifications or training, as well as methodological blind spots. It is also a massive risk. The NTSB documented in Minneapolis in 2007 that standard software and engineering practice at the time did not adequately account for gusset plates; inspections paid little attention to them. This represents a combination of a methodological gap and a competency risk [73,74].

## 5. Discussion and Conclusions

The findings underscore a central tension: advanced SHM technologies demonstrably enhance situational awareness and reduce intervention times, yet they introduce tightly coupled cyber–physical dependencies that can degrade overall resilience if not governed coherently. Treating technical reliability and cybersecurity as separate workstreams obscures joint failure modes—e.g., sensor drift masked by data-lake preprocessing or GNSS spoofing propagating into Digital Twin states and maintenance decisions. A unified risk lens, with shared metrics and decision thresholds, is therefore essential. As the most serious deficiencies were identified:

- Integrated assessment framework: Scientific models addressing both technical reliability and cyber risks together are lacking.
- End-to-end system testing: Projects like CYBRBridge are still in pilot stages; there is a lack of large-scale validation across diverse geographies.
- Regulatory framework: National and international standards for deploying UAVs, IoT, and AI in SHM are incomplete.
- Economic analysis: Cost-benefit assessments of SHM technologies at different stages of the bridge life cycle are missing.

Advanced technologies in SHM are qualitatively advancing bridge monitoring, enabling continuous sensing, automation, and timely intervention. At the same time, they increase system complexity and expose infrastructure to new risks such as cybersecurity, reliability, legal issues depending on laws and regulations, and economic issues. A significant opportunity in research and implementation lies in developing holistic approaches that address these challenges in an integrated way.

Given the increasing use of advanced technologies in bridge monitoring and the identified associated risks, future research should focus on the following key areas:

- Development of a Comprehensive Risk Assessment Methodology for the resilience of (critical) infrastructure currently, there is no unified framework that would allow for systematic assessment and comparison of risks arising from the implementation of technologies such as SHM, IoT, UAVs, or AI. It is proposed to develop a multidisciplinary methodology that integrates technical, organizational, legal, and cybersecurity risks into a single evaluation tool. This tool should be especially useful for critical infrastructure managers and bridge administrators. That requires a holistic approach to the whole problem.
- Testing System Resilience on Real Models and Scenarios is a field where future research should focus on creating realistic simulation scenarios, such as sensor network power outages, data integrity breaches, or AI model misinterpretations. The aim is to verify the systems’ ability to withstand critical situations and propose measures to enhance their resilience. Digital twins, laboratory testing platforms, and digital cybersecurity testbeds can be used for this purpose as well.
- Framework for Legal and Ethical Evaluation The collection and analysis of data via UAVs, sensors, and machine learning algorithms raises concerns regarding GDPR, liability, and ethics. A legal-ethical framework is proposed, which would include:
  ▪ analysis of privacy impacts,
  ▪ clear definition of responsibility in case of system failure,
  ▪ assessment of the transparency of AI decision-making in infrastructure safety contexts.
  ▪ Integration of Technologies: AI, BIM, and Digital Twins
- Research is recommended to focus on the integration of existing technologies, for example, the creation of a platform combining BIM (Building Information Modeling), a digital twin of the assets, and artificial intelligence algorithms. The outcome could be an efficient visualization and decision-making environment that supports maintenance planning, defect detection, and simulations of future structural conditions.
- Socio-technical Acceptance of Technologies in Infrastructure is another crucial part. Even the most advanced system is ineffective if users do not trust it. Future research should therefore explore the level of trust among infrastructure managers and the public toward automated systems. This includes designing training and educational programs for technicians and crisis managers to improve the acceptance of new approaches.

The paper presents an overview article focusing on the risks associated with advanced bridge monitoring technologies (SHM/IoT, UAV, AI, Digital Twin, and cloud platforms). Its greatest added value is that it not only describes the technologies but also frames them through a “monitoring chain” (data collection → visual inspection → intelligent diagnostics → preventive measures) and then links them to risk categorization. The document offers three main types of outputs for future research: a conceptual structure (how to consider risks across the entire monitoring chain), a practical taxonomy of risks divided into technical, cybersecurity, legislative-ethical, and organizational-operational areas.

In terms of the document’s limitations, it is important to note that the technical information is in many places an overview and conceptual, not an experimentally “unified” single verified end-to-end implementation. This means that interactions between sensor errors (drift, failures), data preprocessing, AI models, and DT updates are discussed as real risk mechanisms but are not quantified by a single validation study within a single system. Finally, the document itself acknowledges that regulations, standards, cyber threats, and AI practices are evolving rapidly; therefore, some of the technical and legal-ethical claims are time-sensitive and will require ongoing updates.

## Figures and Tables

**Table 1:** Typical hardware cost and its disadvantages.

Sensor	Costs	Disadvantages
MEMS accelerometer (low-cost)	€100–300	Limited accuracy vs. piezo; noisy; needs many sensors for full coverage
Industrial accelerometer (piezo)	€500–2000	Higher cost per unit; requires cabling and DAQ, power needs
Strain gauge (foil)	€20–200 (per gauge) + €200–1000 per channel	Only local measurement; sensitive to installation quality, temperature compensation required
LVDT displacement transducer	€200–1000	Small measurement range; needs fixed reference, vulnerable to dust/water
Laser displacement sensor	€1000–5000	Expensive, sensitive to weather/dust, requires precise alignment
Robotic total station	€20,000–40,000	Line of sight required, cannot capture fast dynamics, and is a costly instrument
GNSS station	€10,000–20,000	Poor short-term dynamic accuracy, expensive per point, requires a clear sky
Fiber-optic (FBG/DFOS)	€15,000–50,000 (interrogator) + €10–30/m fiber	Very high capex; fragile fibers, complex installation and repairs
Acoustic emission sensor	€1000–3000	Data interpretation is complex, prone to false positives, and has only local coverage
Corrosion probe	€500–2000	Local information only, limited probe lifespan; calibration issues
Weigh-in-Motion	€30,000–50,000 per lane	Only measures load, not conditions, expensive, requires roadworks
Vision/Camera (high-res)	€300–8000	Sensitive to lighting/weather, requires stable mounting and calibration, huge data processing/storage needs

**Table 2:** SWOT analysis for the risks arising from the use of advanced technologies in the monitoring of bridges.

Strenghts	Importance	Rating	Weaknesses	Importance	Rating
High accuracy in data collection and evaluation	0.3	5	High initial and operational costs	0.4	5
Realtime monitoring of structural conditions	0.3	4	Need for qualified personnel and IT infrastructure	0.3	2
Support for predictive maintenance and failure prevention	0.2	3	Possibility of malfunctions and false alarms	0.15	2
Integration with intelligent infrastructure (smart cities)	0.2	3	Dependence on specific technology providers	0.15	1
Result sum	**3.9**		Result sum	**3.05**	
**Oportunities**	**Importance**	**Rating**	**Threats**	**Importance**	**Rating**
Development of smart infrastructure and digital twins	0.4	5	Cyberattacks and data manipulation	0.5	4
Funding opportunities for research and innovation	0.25	2	Legal and ethical issues (GDPR, …)	0.2	2
Improved crisis response and preventive measures	0.25	2	Rapid obsolescence of technology	0.2	2
International standardization and cooperation	0.1	2	Insufficient regulation or unclear responsibilities	0.1	2
Result sum	**3.2**		Result sum	**3**	

**Table 3:** Risk Matrix.

Category	Monitoring Chain	Risk	P (1–5)	I (1–5)	R	Priority
Technical	Data acquisition	Sensor or device failure	4	4	**16**	**High**
Technical	Intelligent diagnoses	Low accuracy of AI models in real-world conditions	3	4	**12**	Medium
Technical	Data acquisition	Misinterpretation of data	3	3	**9**	Medium
Technical	Data acquisition/Visual inspection	Technological obsolescence/incompatibility	4	3	**12**	Medium
Cybersecurity	Intelligent diagnoses	Hacking and unauthorized access (Data manipulation)	3	5	**15**	**High**
Cybersecurity	Data acquisition/Intelligent diagnoses	Insufficient encryption during transmission	3	4	**12**	Medium
Cybersecurity	Data acquisition	Dependance on the internet/cloud (Connection loss)	3	4	**12**	Medium
Legal/Ethical	Visual inspection	GDPR violations (UAV near residential areas)	3	4	**12**	Medium
Legal/Ethical	Intelligent diagnoses/Preventive measurements	Unclear liability in case of system failure	3	4	**12**	Medium
Legal/Ethical	Preventive measurements	Lack of standards and guidelines in construction	3	3	**9**	Medium
Organizational Operational	Data acquisition/Preventive measurements	Lack of qualified personnel	4	4	**16**	**High**
Organizational Operational	Preventive measurements	Poor integration with maintenance processes	4	3	**12**	Medium
Organizational Operational	Data acquisition/Preventive measurements	Vendor lock-in (specific provider/software)	3	3	**9**	Medium

**Table 4:** Risk with endangered areas.

Area	Risk
Privacy	Unauthorized collection of sensitive data
AI models	Biased data, lack of transparency
Safety	Incorrect condition assessment, risk of structural failure
Social impacts	Job losses, unequal distribution of benefits
Abuse	Use of drones for violence

## Data Availability

No new data were created or analyzed in this study. Data sharing is not applicable to this article.
